# Monoclonal antibodies raised against the Junín virus glycoprotein show broad reactivity and provide protection *in vivo*

**DOI:** 10.1128/mbio.00214-26

**Published:** 2026-09-25

**Authors:** Garazi Pena Alzua, Sharon Jan, Fatima Amanat, Grecia A. Sandoval, Christopher A. Sumner, Stephen Won, Anna L. Lara, Anupama Sinha, Brooke N. Harmon, Elizabeth B. Allmon, Kendra J. Alfson, Yenny Goez-Gazi, John W. Dutton, Ricardo Carrion, Sneha Rangarajan, Melissa C. Kerzic, Carter A. Mitchell, Chris M. Cirimotich, Dylan M. Johnson, Florian Krammer

**Affiliations:** 1 Department of Microbiology, Icahn School of Medicine at Mount Sinai 5925, New York, New York, USA; 2 Center for Vaccine Research and Pandemic Preparedness (C-VaRPP), Icahn School of Medicine at Mount Sinai 5925, New York, New York, USA; 3 Department of Biotechnology & Bioengineering, Sandia National Laboratory 1105, Livermore, California, USA; 4 Texas Biomedical Research Institute 7075, San Antonio, Texas, USA; 5 Southwest National Primate Research Center, San Antonio, Texas, USA; 6 Kemp Proteins, LLC, Frederick, Maryland, USA; 7 Battelle Biomedical Research Center 125990, West Jefferson, Ohio, USA; 8 Department of Biosecurity & Bioassurance, Sandia National Laboratory 1105, Livermore, California, USA; 9 Department of Pathology, Molecular and Cell-Based Medicine, Icahn School of Medicine at Mount Sinai 5925, New York, New York, USA; 10 Ignaz Semmelweis Institute, Interuniversity Institute for Infection Research, Medical University of Vienna 27271, Vienna, Vienna, Austria; 11 Ludwig Boltzmann Institute for Science Outreach and Pandemic Preparedness at the Medical University of Vienna, Medical University of Vienna 27271, Vienna, Austria; Duke University School of Medicine, Durham, North Carolina, USA

**Keywords:** Junín virus, glycoprotein complex, monoclonal antibodies, neutralizing antibodies, *in vivo *efficacy

## Abstract

Argentine hemorrhagic fever (AHF) is a disease caused by the Junín virus (JUNV) that can be fatal in 15%–30% of untreated cases. Although JUNV is the only arenavirus for which efficient therapy and vaccination have been established, its administration is limited. In this study, hybridoma technology was used to generate 30 mouse monoclonal antibodies (mAbs) against the glycoprotein complex (GPC) of JUNV. Of these 30 mAbs, 26 showed strong cross-binding to GPCs from various arenaviruses. In addition, nine mAbs demonstrated potent neutralizing activity *in vitro* with a 50% inhibitory concentration of less than 1 µg/mL against a replication-competent chimeric vesicular stomatitis virus expressing JUNV GPC (rVSV-JUNV), as well as the live attenuated JUNV vaccine strain Candid#1. Furthermore, when tested *in vivo* using a challenge model of rVSV-JUNV in Stat2^−/−^ mice, five select mAbs significantly reduced viral loads in the spleen when administered prophylactically. Humanized versions of JUNV-1A2, JUNV-1D5, and JUNV-5B12 mAbs, which showed protection *in vivo* in the Stat2^−/−^ surrogate model, were produced. These antibodies were evaluated in a guinea pig challenge model of AHF and showed protective efficacy. Escape experiments with these humanized antibodies, as well as previously characterized antibodies, GB03 and JUN1, using rVSV-JUNV resulted in the acquisition of amino acid changes in the GP1 part of the GPC, which directly contacts host receptor transferrin receptor 1, indicating these sites could be key for future therapies. In summary, we generated mAbs that may be valuable for antibody-guided vaccine design and therapeutic development.

**IMPORTANCE:**

Junín virus (JUNV) is a highly pathogenic virus that causes Argentine hemorrhagic fever (AHF) and is classified as a category A pathogen due to its potential for aerosol transmission and the significant number of annual infections and deaths associated with it. Although effective treatments exist, such as convalescent immune plasma, their availability is limited, and they are ineffective when given after the first week of symptoms. This study is significant because it focuses on developing therapeutic monoclonal antibodies against JUNV with strong *in vitro* neutralizing activity and protection in both a surrogate mouse model and a guinea pig challenge model of AHF. This has led to promising antibody candidates that could be used as therapeutics to combat ongoing outbreaks.

## INTRODUCTION

New World (NW) arenaviruses are rodent-borne enveloped RNA viruses, transmitted through inhalation of aerosolized body fluids or excretions from infected rodents. Among these pathogenic arenaviruses, the Junín virus (JUNV), an NW arenavirus, causes Argentine hemorrhagic fever (AHF) in humans, with outbreaks occurring during the harvest season in endemic areas (1). The endemic zone is expanding, with 20%–30% of infected individuals developing hemorrhagic or neurological complications, and the case-fatality rate ranges between 15% and 30% if untreated (2–4). Due to these facts and other reasons, JUNV is considered a serious public health concern. Convalescent plasma is an effective treatment for individuals infected with JUNV. However, studies have reported late-onset neurological syndrome in both humans and experimental animals after convalescent plasma treatment (5–7). The Candid#1 vaccine, a live attenuated JUNV vaccine licensed in Argentina (8), has markedly reduced the incidence of AHF. However, it is important to note that the U.S. Food and Drug Administration has not approved this vaccine; it only provides protection against one arenavirus serogroup and has been associated with adverse events (4, 8, 9).

The arenavirus genome consists of two ambisense RNA segments, designated as large and small (10). The small segment encodes the structural proteins, including the nucleoprotein and the glycoprotein precursor complex (GPC) embedded on the surface of the mature virion (11). Post-translational cleavage by host proteases yields two glycoprotein subunits, GP1 and GP2, which, together with a stable signal peptide, form the GPC trimer. This trimer represents the functional form of the envelope protein that mediates virion attachment and fusion with host membranes (12). JUNV, along with other pathogenic NW arenaviruses, is thought to use the cellular transferrin receptor 1 (TfR1) to infect cells (13). GPC not only plays an important role in cell entry but is also the main target for neutralizing antibodies (14–18), which are considered the main immune defense against infection. GPC is an attractive target for vaccine development, with the potential to induce cross-reactive antibodies against different arenaviruses (19).

In this study, 30 monoclonal antibodies (mAbs) were generated using murine hybridoma technology (19, 20). The process involved fusing spleen cells from mice immunized with DNA encoding the GPC and recombinant JUNV GPC with myeloma cells, resulting in the generation of mAbs that bind the GPC of various arenaviruses and confer protection *in vivo* in a surrogate challenge model. Furthermore, humanized versions of the characterized antibodies were generated to test their therapeutic potential through pharmacokinetic analysis and *in vivo* protection assays in guinea pigs.

## RESULTS

### Generated mAbs are cross-reactive to the GPC of several NW and Old World arenaviruses, effectively neutralize JUNV *in vitro*, and show activity in an antibody-dependent cell-mediated cytotoxicity reporter assay

**Fig 1:**
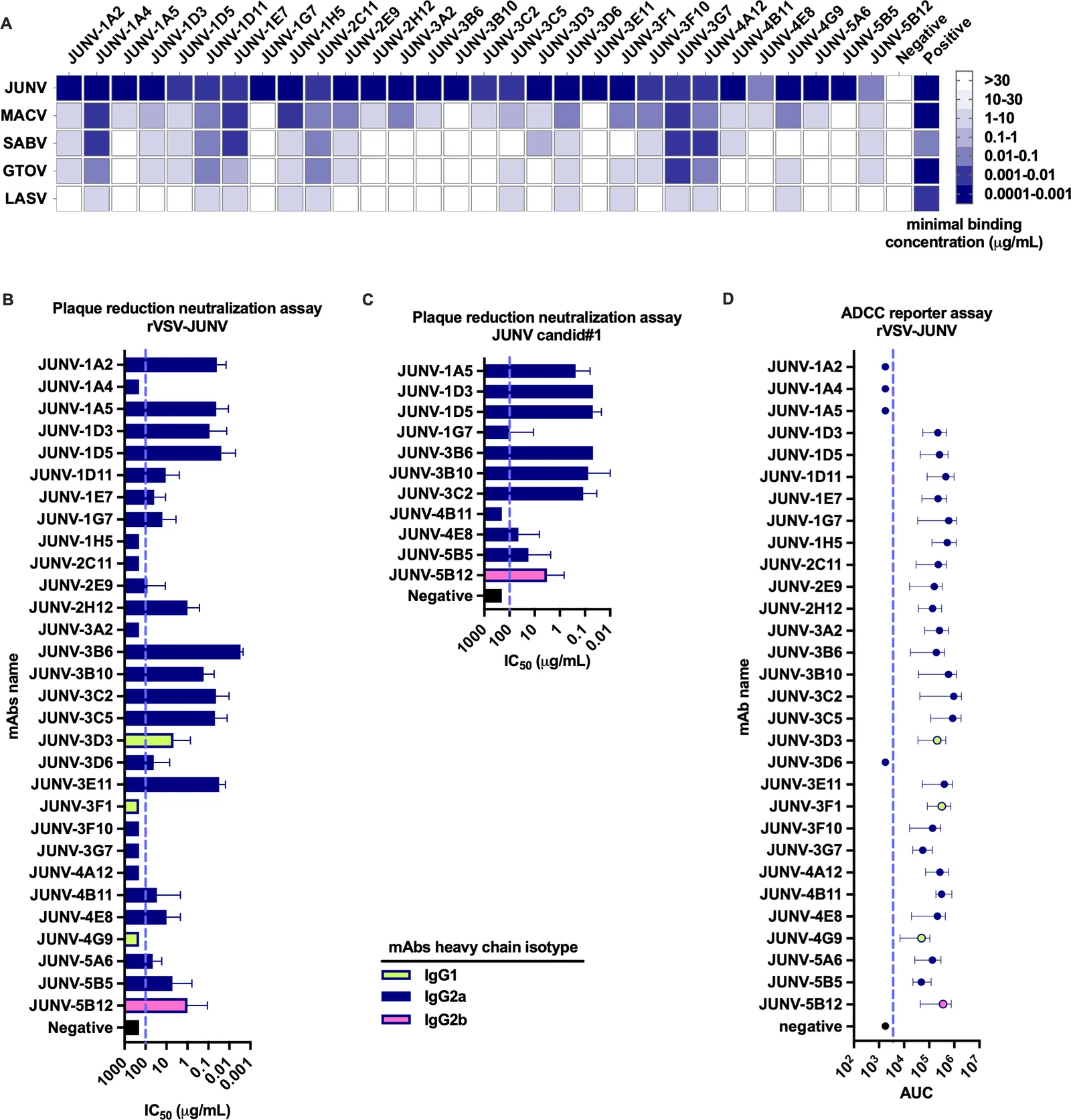
mAbs binding to the glycoprotein precursor complex (GPC) of different arenaviruses, along with their functional characterization by *in vitro* neutralization and ADCC reporter assay. (**A**) Binding capacity of all 30 mAbs against different GPCs of NW arenaviruses (JUNV, MACV, SABV, and GTOV) and of an OW arenavirus (LASV) was tested using ELISA. Each antibody was tested at a starting concentration of 30 µg/mL, followed by a 1:3 serial dilution. Data are shown as minimal binding concentrations, the lowest concentration at which a signal is observed. Neutralizing activity was tested against (**B**) rVSV-JUNV and (**C**) JUNV Candid#1, at a starting concentration of 100 µg/mL in duplicate using VeroE6 cells. Murine anti-JUNV antibodies are color-coded by heavy-chain isotype. IC_50_ values with 95% confidence intervals are shown, calculated with GraphPad Prism using top and bottom constraints of 0% and 100%. The line indicates the maximum tested concentration without neutralization; negatives are assigned half this value. (**D**) ADCC reporter assay measured antibody-dependent cell-mediated cytotoxicity via luciferase activity using rVSV-JUNV-infected VeroE6 cells co-cultured with FcγRIV effector cells; results shown as area under the curve (AUC), calculated with GraphPad Prism 11.

A hybridoma fusion approach was used to induce antibodies against JUNV GPC. This involved fusion of the spleen cells from mice immunized with a plasmid encoding the JUNV GPC, followed by a boost with a recombinant JUNV GPC protein produced using the baculovirus expression system (19, 20). This resulted in the isolation of 30 IgG-secreting hybridoma clones against the JUNV GPC. The antibodies from individual clones were purified, and the binding breadth of all 30 mAbs was assessed using an enzyme-linked immunosorbent assay (ELISA) (Fig. 1A). In order to evaluate the cross-binding capacity of the generated mAbs, the GPC of five different arenaviruses belonging to both NW and Old World (OW) serogroups was used. The assay included the GPCs of JUNV, Machupo virus (MACV, NW) (21), Sabiá virus (SABV, NW) (22), Guanarito virus (GTOV, NW) (23), and Lassa virus (LASV, OW) (24). Of the 30 generated mAbs, 26 showed strong binding to more than one GPC from different viruses, and are considered broadly cross-reactive antiarenavirus GPC antibodies. These 26 mAbs exhibited two different binding patterns. The first pattern was observed with eight antibodies (1A4, 1D11, 1E7, 1H5, 2C11, 3D6, 3G7, and 4A12), which showed strong binding to all tested recombinant GPCs, including GPC from both NW and OW arenaviruses. The second pattern was observed with six mAbs (1A2, 1D3, 1D5, 2E9, 3F10, and 5B12), which bound to all NW arenaviruses but not to LASV, belonging to the OW arenaviruses. The remaining mAbs showed different binding tendencies. Of note, only four mAbs did not bind to the JUNV-related MACV GPC.

The functionality of these antibodies was evaluated by initially testing their neutralization capacity using an *in vitro* plaque reduction neutralization assay (PRNAs) with recombinant vesicular stomatitis virus (rVSV) expressing JUNV GPC, here referred to as replication-competent chimeric vesicular stomatitis virus expressing JUNV GPC (rVSV-JUNV) (Fig. 1B). Of the 30 generated mAbs, 20 neutralized rVSV-JUNV. Among these, nine mAbs (1A2, 1A5, 1D3, 1D5, 3B6, 3B10, 3C2, 3C5, and 3E11) had a 50% inhibitory concentration (IC_50_) below 1 µg/mL, suggesting potent neutralization capacity. Antibodies were also isotyped, and most were IgG2a; three were IgG1 (3D3, 3F1, and 4G9), and mAb 5B12 was the only IgG2b. Overall, IgG1 antibodies showed little to no neutralizing activity against rVSV-JUNV (Fig. 1B). Eleven antibodies, including both rVSV-JUNV neutralizers and non-neutralizers, were also tested against the JUNV vaccine strain Candid#1. The same mAbs that neutralized rVSV-JUNV also neutralized Candid#1 with comparable IC_50_ values, and non-neutralizers showed no activity against Candid#1 (Fig. 1C). Previous studies have shown that interaction between the Fc region of antibodies with Fc gamma receptors (FcγRs) present on effector cells, like natural killer cells or macrophages, can lead to protection *in vivo* (25, 26). Furthermore, recent studies have demonstrated that antibodies against NW arenaviruses that efficiently activate Fc receptors *in vitro* conferred protection *in vivo* (20). In this study, an antibody-dependent cellular cytotoxicity (ADCC) reporter assay was used to investigate the Fc-mediated effector function of the generated mAbs, and the interaction between the Fc and FcγR was determined for rVSV-JUNV-infected cells (Fig. 1D). Both neutralizing and non-neutralizing antibodies showed ADCC activity. Interestingly, the mAbs (1A2 and 1A5) which had high neutralizing capacity, demonstrated the lowest or no ADCC activity, alongside one weak neutralizer (3D6). On the other hand, the mAbs (1G7 and 1H5), which had low or non-neutralizing capacity, demonstrated high ADCC activity, with mAbs (3B10, 3C2, and 3C5) displaying the highest ADCC capacity.

### Administration of generated mAbs reduces JUNV viral titers in a surrogate rVSV-JUNV challenge Stat2^−/−^ mouse model

**Fig 2:**
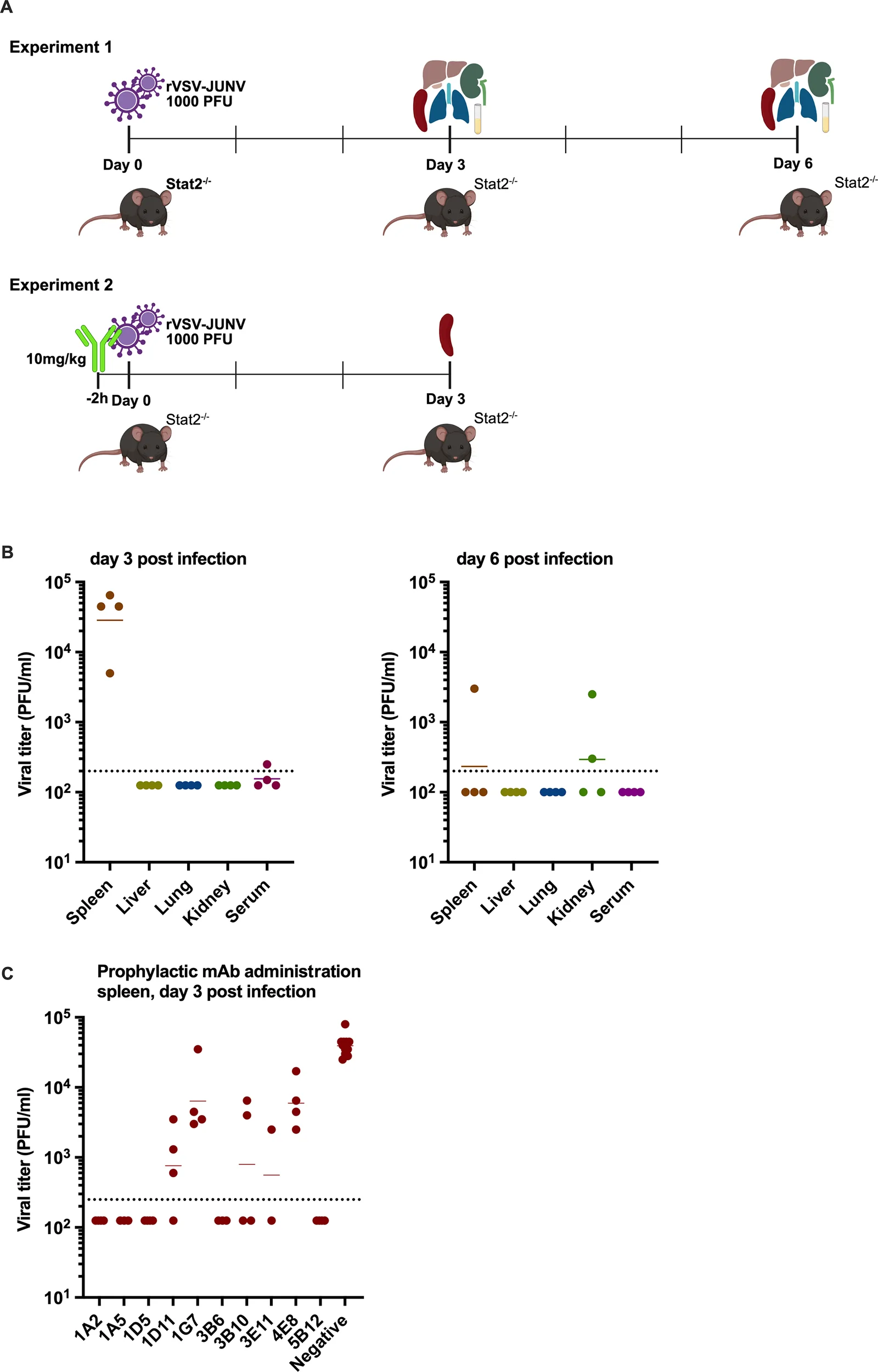
Generated mAbs confer protection against rVSV-JUNV infection *in vivo*. (**A**) Overview of the experiments performed in Stat2^−/−^ mice. The first timeline corresponds to panel** B**, and the second timeline corresponds to panel **C**. (**B**) Viral loads in harvested organs (spleen, liver, lung, and kidney) were measured, and samples were collected on days 3 and 6 after infection with rVSV-JUNV. (**C**) A prophylactic study was conducted by administering 10 mg/kg of each mAb intraperitoneally in Stat2^−/−^ mice, followed by intraperitoneal infection with 1,000 plaque-forming units (PFU) of rVSV-JUNV. Spleens from individual mice were harvested on day 3 post-infection to measure viral titers. (**B and C**) Viral titers (PFU/mL) are shown, with each dot representing an individual mouse virus titer, and the geometric mean is indicated. The dashed line on the *y*-axis marks the limit of detection, defined as half the lowest dilution with a positive assay response.

In the past, we have used chimeric rVSV expressing different viral glycoproteins to challenge Stat2^−/−^ mice. These models, while not bona fide challenge models with the respective viruses, do allow the determination of protective effects of mAbs against the glycoproteins *in vivo* in a lower biocontainment environment (20, 27). To test this system with rVSV-JUNV, we infected Stat2^−/−^ mice with 1,000 plaque-forming units (PFU) of virus and harvested spleen, liver, lung, kidney, and serum on days 3 and 6 (Fig. 2A). On day 3, we found high viral titers in the animals’ spleens (approximately 3 × 10^4^ PFU/mL of organ suspension). Little to no viral replication was observed in other organs or on day 6 post-challenge (Fig. 2B). We then tested the effectiveness of selected mAbs in conferring protection against rVSV-JUNV infection in this model using spleen titers on day 3 post infection as readout (Fig. 2C). The test involved four types of mAbs: (i) potent neutralizing mAbs (1A2, 1A5, 1D5, 3B6, 3B10, and 3E11); (ii) a mAb that cross-binds to the GPC of all different arenaviruses (1D11); (iii) low neutralizing mAbs with high ADCC activity (1G7 and 5B12); and (iv) a mAb with low neutralizing and low ADCC activity (4E8). Our main objective was to determine which mAbs could provide the best protection against JUNV infection *in vivo*. For that, Stat2^−/−^ mice were infected with 1,000 PFUs of rVSV-JUNV, and at day 3 post-infection, the spleen was harvested, and viral titers were determined. Out of the ten mAbs, five (1A2, 1A5, 1D5, 3B6, and 5B12) effectively reduced viral titer in mice when given prophylactically at a dose of 10 mg/kg body weight. The reduction in viral titer was primarily attributed to potent neutralizing antibodies, with the exception of mAb 5B12, which showed robust protection despite lower *in vitro* neutralizing activity.

### Humanized antibodies show high affinity to JUNV GPC and efficiently neutralized JUNV *in vitro*

**Fig 3:**
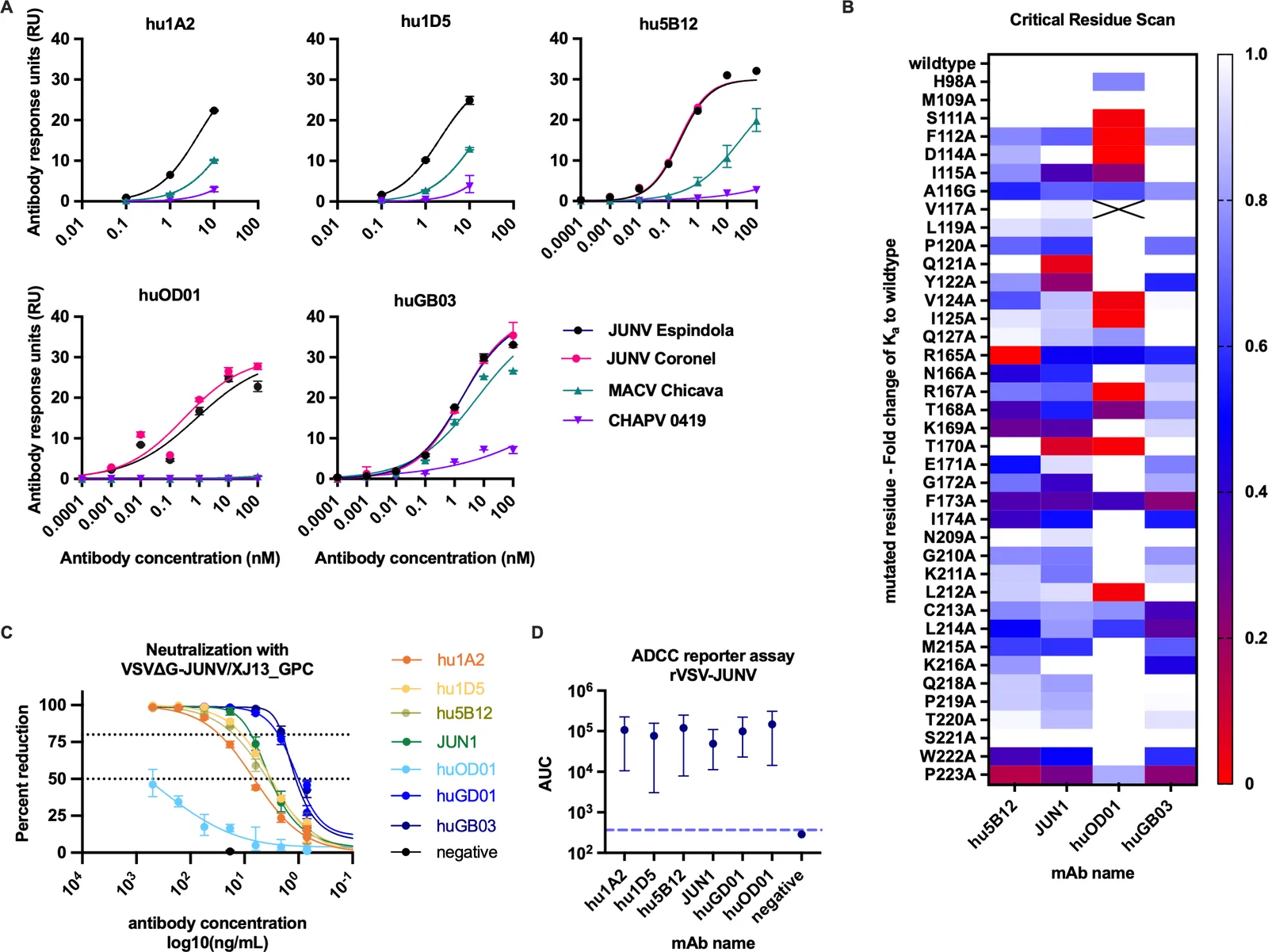
Production of humanized antibodies and assessment of their pharmacokinetics and neutralization activity *in vitro*. (**A**) Humanized anti-JUNV mAbs 1A2, 1D5, and 5B12 with human IgG1 constant regions were generated. The binding affinity to different arenaviruses and non-linear regression based on a four-point sigmoidal curve was generated using GraphPad Prism 11. (**B**) Biolayer interferometry-based fold change in *K*_*a*_ compared to wild-type JUNV/Espindola sGP1 of humanized mAbs against a selective alanine mutant library (as indicated on the *y*-axis). (**C**) An *in vitro* neutralization assay was performed to test the mAbs’ ability to neutralize VSVΔG-JUNV/XJ13 in duplicate, and values are reported as the percent reduction in FFU detected compared to antibody-negative controls. A non-linear regression based on the inhibitory concentration versus normalized response {*Y* = 100/[1 + (IC_50_/*X*)Hill slope]} was used to generate the curves in GraphPad. Dashed lines on the *y*-axis indicate the 50% and 80% inhibitory concentrations. (**D**) ADCC reporter assay measured antibody-dependent cell-mediated cytotoxicity via luciferase activity using rVSV-JUNV-infected VeroE6 cells co-cultured with FcγRIIIa effector cells; results are shown as area under the curve (AUC), calculated with GraphPad Prism 11.

Following the initial characterization of the murine mAbs and showing their successful protective capacity *in vivo*, humanized versions—chimeric antibodies with murine VH/VL regions in human IgG1 and Igκ backbones of JUNV-1A2, JUNV-1D5, JUNV-5B12, OD01, GD01, and GB03 (16, 28) antibodies, referred to as hu1A2, hu1D5, hu5B12, huOD01, huGD01, and huGB03, were developed and evaluated for potential therapeutic use. The Gyroslab miniaturized ELISA bioanalytical platform was used to analyze the binding of humanized IgGs. These engineered antibodies, which contain the variable regions of the native murine light and heavy chains with the human IgG1κ constant region, were tested against the soluble receptor-binding domain (sGP1) portion of GPC from the JUNV Espindola and Coronel strains, the MACV Chicava strain, and the Chapare virus (CHAPV) 810419 strain. All of these antibodies showed high-affinity binding to the JUNV Espindola glycoprotein, with half-maximal binding below 10 nM. Similar responses were observed with the evolutionarily distant JUNV Coronel strain. A weaker response was observed against the MACV Chicava glycoprotein, except for huOD01, which did not bind. None of the antibodies were reactive to the glycoprotein of CHAPV 810419, except for a weak interaction detected with huGB03 (Fig. 3A). No response was observed with an irrelevant human IgG1κ isotype mAb (not shown), which served as a negative control. Alanine mutants across the common antibody-binding region of JUNV Espindola sGP1 were generated, and antibody-binding affinity was compared to wild-type sequences using biolayer interferometry (Fig. 3B). The binding of hu5B12 was diminished by R165A, K169A, and P223A mutants; huGB03 binding was diminished by F173A, L214A, and P223A; huOD01 binding was diminished by S111A, F112A, D114A, I115A, V124A, I125A, R167A, T170A, and L212A; and huJUN1 binding was diminished by Q121A, Y122A, T170A, F173A, and P223A.

The humanized antibodies were tested in an *in vitro* neutralization assay against rVSVΔG-JUNV/XJ13_GPC (Fig. 3C). Humanized JUNV mAbs, hu1D5 (29.63 ng/mL IC_50_), hu5B12 (33.93 ng/mL IC_50_), hu1A2 (64.96 ng/mL IC_50_), huGB03 (7.19 ng/mL IC_50_), huGD01 (6.26 ng/mL IC_50_), and JUN1 (28.96 ng/mL IC_50_) were all effective at neutralizing JUNV, with IC_50_ values comparable to previously reported values for JUN1, GD01, and GB03 (16). These antibodies target the glycoprotein 1 (GP1) domain of the JUNV protein, which is crucial for viral entry into host cells by binding to the human transferrin receptor 1 (hTfR1). By binding to TfR1, these antibodies block the GPC-TfR1 interaction, resulting in virus neutralization (29). However, OD01 was not completely neutralizing at the tested concentrations. Additionally, hu5B12, hu1D5, and huGB03 showed no neutralization of rVSVΔG-MACV/Carvallo_GPC at concentrations up to 5 μg/mL. Furthermore, hu5B12 and hu1D5 also did not neutralize rVSVΔG-CHAPV/810419_GPC at the same concentration. An ADCC assay was performed to evaluate the Fc-mediated effector function of the chimeric antibodies. The interaction between the antibody Fc region and human FcγRIIIa was assessed with rVSV-JUNV-infected cells (Fig. 3D). All tested antibodies exhibited comparable activity *in vitro*.

### Administration of human mAbs provides full protection in guinea pigs infected with the JUNV Romero strain

**Fig 4:**
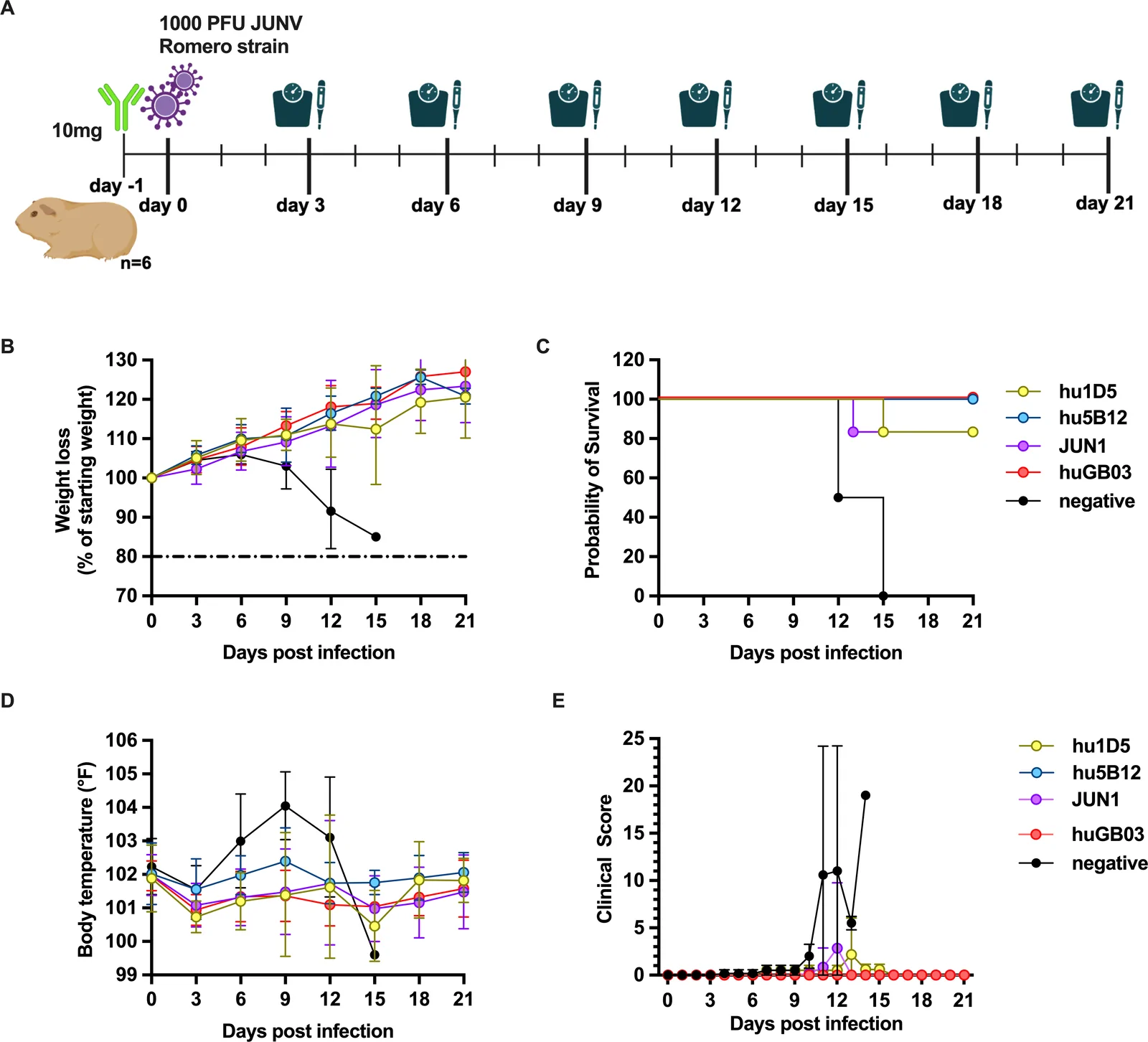
Protection was observed after mAbs administration in guinea pigs infected with the JUNV Romero strain. (**A**) Overview of the guinea pig experiment. Every 3 days post-infection, body weight percentage (**B**), survival (**C**), and body temperature (**D**) were collected and plotted over 21 days. Body weight percentage is relative to the starting body weight, and the data are presented as geometric means with standard deviations. The dashed line at the *y*-axis indicates the ethical endpoint. Clinical score (**E**) was monitored daily, scoring signs like dyspnea, responsiveness, discoordination, grooming, posture indicating signs of pain or distress (e.g., hunched back), and eye appearance (e.g., closed or partially closed). Clinical scores greater than or equal to 12 meet euthanasia criteria.

The outbred Hartley guinea pig model mimics most clinical symptoms of AHF, such as fever, leukopenia, thrombocytopenia, hemorrhagic signs, elevated liver enzymes, and death around 14 days after infection, making it a suitable model for preclinical research. Female Hartley guinea pigs were divided into groups of six (*n* = 6). Each group received an intraperitoneal injection of 10 mg (~40 mg/kg) of hu1D5, hu5B12, huJUN1, or huGB03 mAbs. A control group received only the vehicle. The next day, all animals were challenged with 1,000 PFU of the Romero strain of Junín virus (Fig. 4A) and were monitored for clinical signs, weight loss, and temperature over 21 days post-infection. The antibodies hu1D5 and hu5B12 (first described here), along with JUN1(17) and huGB03 (16) mAbs targeting specific sites on GP1 and used therapeutically, showed protective effects in guinea pigs with significantly increased survival compared to mock-treated animals (Mantel-Cox log-rank test, *P* < 0.0001), although there were no significant differences in survival among these mAb-treated groups. In contrast, guinea pigs receiving only the vehicle showed significant weight loss by day 15 post-infection (Fig. 4B). Notably, hu5B12 and huGB03 provided complete protection against JUNV infection in guinea pigs compared to other groups (Fig. 4C). Similar to weight loss, untreated animals showed marked temperature drops by day 15 (Fig. 4D). Daily monitoring included clinical signs like tremors, loss of balance, paralysis, and hemorrhage. Guinea pigs treated with hu5B12 and huGB03 showed no clinical signs. Consistent with the data on weight loss and survival, vehicle-treated guinea pigs exhibited AHF symptoms from days 9 to 15, reaching the ethical endpoint by day 15. The guinea pig that died after being administered with JUN1 antibody showed symptoms on day 12 post-infection, while the one in the hu1D5 group showed symptoms on day 13.

### Antibody pressure in cell culture leads to amino acid changes in the receptor-binding site of GPC

**Fig 5:**
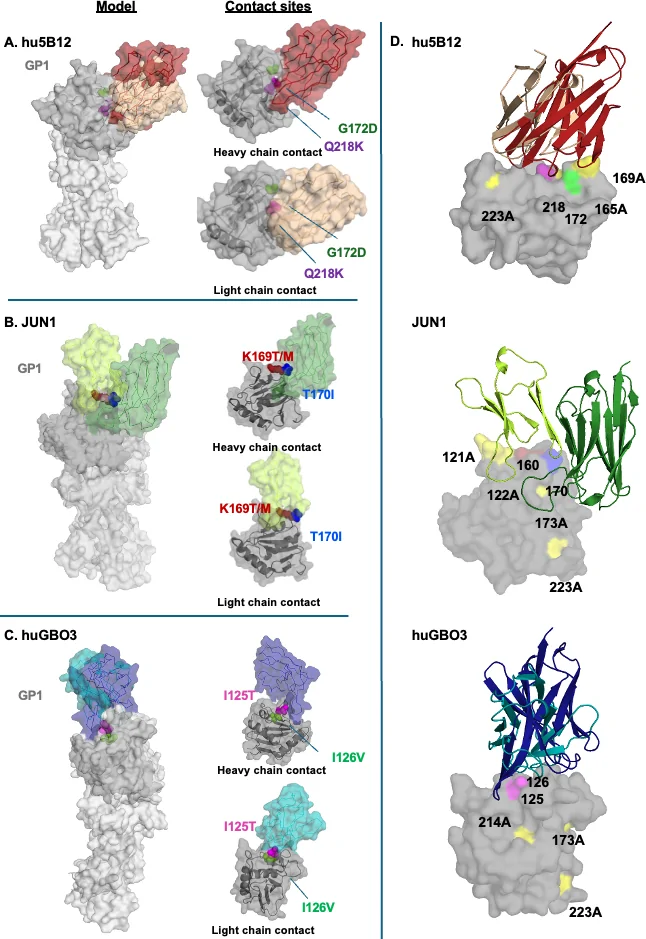
Antibody-driven immune pressure led to the emergence of escape mutants in JUNV GP1. rVSVΔG-JUNV/XJ13 GPC was cultured with (**A**) hu5B12, (**B**) JUN1, or (**C**) huGB03 antibodies. After seven passages, viral RNA was extracted and the sequence analyzed. The final full-length Chai-1 model (32, 33) is shown in lighter gray, with the GP1 region in darker gray. Protein structures and identified mutations were visualized using PyMOL (The PyMOL Molecular Graphics System, version 3.0, Schrödinger, LLC), showing heavy chains in darker and light chains in lighter shades. Contact points with both chains, as well as the position of the amino acid changes in GP1, are shown next to the protein model. (**D**) Mutated sites from the alanine scanning experiment (Fig. 3B) that showed reduced binding with the indicated mAbs. Co-folding prediction of GP1 with heavy and light chains of hu5B12, JUN1, and huGB03 was performed. Mutations introduced to an alanine are highlighted in yellow, with escape mutants color-coded as in panels **A–C**.

rVSVΔG-JUNV/XJ13 GPC was incubated with hu5B12, JUN1, or huGB03 antibodies in Vero cells for a total of seven passages, starting with the 80% focus reduction neutralization titer (FRNT80) calculated based on neutralization of reporter rVSVΔG-JUNV/XJ13_GPC (33.9, 29.0, and 7.2 ng/mL for hu5B12, JUN1, and huGB03, respectively) and ending at a final concentration 64 times the FRNT80. Post-passage, viral sequence analysis revealed that serial passage with each antibody resulted in escape mutants in the GP1 protein. With hu5B12, two non-synonymous mutations were identified: one at position G172, changing to D, and another at Q218, changing to K (Fig. 5A). Residue G172 lies in loop 7, being a target for neutralizing antibodies (29), and this same loop has been described to protrude from the GP1 core to contact TfR1 (30). In more detail, this mutation has been found to make direct contact with TfR1 (31). When using JUN1 mAb, two mismatch mutations emerged after immune pressure at positions where K169 was substituted to T or M and T170 to I (Fig. 5B). Notably, these residues directly interact with the JUNV receptor, TfR1 (30). The same mutations at these residues were observed in other NW arenaviruses, such as MACV, under specific immune pressure with anti-MACV mAbs (20). Finally, with the huGB03 antibody, two mutations were identified after serial passage; I125 was substituted with T and I126 with V, both of which are near the receptor-binding site (Fig. 5C). These mAbs are predicted to bind to a similar region of GP1, based on the full protein sequence from GenBank accession number AAQ55251 and antibody sequences. Confidence scores from predicted template modeling (pTM) and interface predicted template modeling (ipTM) for 5B12, JUN1, and huGB03, using both heavy and light chains, are shown in Fig. S1 and Table S3. For 5B12, the pTM ranges from 0.65 to 0.72, and the ipTM ranges from 0.27 to 0.58; for JUN1, the pTM is 0.64–0.66, and the ipTM is 0.23–0.28; for GB03, the pTM is 0.64–0.66, and the ipTM is 0.28–0.42.

Interestingly, the same positions or nearby sites of substitution identified after selective immune pressure also show reduced binding in the alanine-scanning experiment (Fig. 3B). For hu5B12, target sites are 165–172 and 218–223 in JUNV GP1; for huJUN1, an amino acid change at position 170 is shared in both experiments; and for huGB03, no similarities were observed. However, the alanine scan with huJUN1 indicated that substitutions at sites 121 and 122 decreased binding; similar sites are seen in the escape experiment with huGB03, where amino acid changes occur at positions 125 and 126 (Fig. 5A through D; Table S2). Because all the positions are within the GP1 protein and a high-resolution structure has been determined (PDB: 5W1K), predicted sites were analyzed using this crystal structure and antibody sequences. Confidence scores were more accurate than when using the full protein sequence (Table S3). For 5B12, the pTM ranges from 0.81 to 0.72, and the ipTM ranges from 0.28 to 0.54; for JUN1, the pTM ranges from 0.67 to 0.78, and the ipTM ranges from 0.29 to 0.31; for GB03, the pTM ranges from 0.66 to 0.72, and the ipTM ranges from 0.25 to 0.33. Overall, results with these three antibodies suggest that positions 165–172, 218–223, and 121–126 are the main target regions on JUNV GP1 for these neutralizing antibodies.

## DISCUSSION

Both OW and NW arenaviruses include examples of medically important pathogens, with JUNV being one of them. Monoclonal antibody therapeutics have recently been shown to be effective in protecting against JUNV disease in different animal models (14, 15) and could, over time, replace currently used convalescent plasma therapy (5–7). Here, we isolated 30 mAbs raised against the JUNV GPC in mice using an immunization strategy similar to the one we previously developed for MACV (20). Of the 30 mAbs, a substantial number (87%) showed cross-reactivity to MACV GPC. This is in sharp contrast to what we have experienced with mAbs raised against MACV GPC, where only 32% cross-reacted to JUNV GPC (20). The two viruses are closely related, but MACV has an insertion of 11 amino acids in the GP1 compared to JUNV (34). Furthermore, a large percentage of antibodies cross-reacted with more distantly related NW arenavirus GPCs or even LASV GPC. About half of the mAbs isolated also had neutralizing activity, neutralizing both rVSV-JUNV and Candid#1 with similar potency. However, most of the cross-reactive mAbs that bind to both NW and OW arenavirus GPC, including 1A4, 1D11, 1E7, 1H5, 2C11, 3G7, and 4C12, did not exhibit neutralizing activity against JUNV *in vitro*. Studies indicate that cross-binding to mature GPC of different arenaviruses mainly occurs in the conserved GP2 region (19, 35), which may explain the lack of neutralization. Another study suggests that neutralization of OW arenavirus requires interaction with GP1, whereas binding to GP2 alone is insufficient for effective neutralization (36). Although MACV is a NW virus closely related to JUNV, sharing structural features and the use of hTfR1 for cell entry, significant sequence and structural differences exist at the receptor-binding site, with more than 48% variation. These differences may hinder cross-neutralization despite the ability of some antibodies to bind shared conserved regions on GP1. JUNV neutralizing antibodies in this study could recognize shared epitopes but lack the steric hindrance or receptor-mimicking interactions necessary for neutralization of both viruses (31, 37). We also detected strong ADCC reporter activity for several of the isolated mAbs, including those with no neutralizing activity. While the importance of Fc-FcR interactions for many viral infections has been established, their role in protection from JUNV remains unknown, and therefore, we cannot determine the significance of this finding.

Next, we used a surrogate challenge model to determine whether a selection of the generated mAbs could protect *in vivo*. This model is artificial since it uses a replication-competent VSV expressing the JUNV GPC to infect Stat2^−/−^ mice. However, it has the advantage of lower biocontainment requirements as compared to authentic JUNV, and the chimeric VSV relies on the JUNV GPC for replication, making it a useful *in vivo* model of antibody-mediated neutralization, which potentially recapitulates limited (due to the Stat2^−/−^ and resulting defects in interferon-based immunity) ADCC. In this model, we found that, in general, strong neutralizing antibodies reduced virus replication most potently, but one low neutralizing antibody also blocked virus replication completely. Of note, all tested antibodies had an effect in the model compared to the appropriate negative control antibody. We have used this system successfully before to test the *in vivo* efficacy of MACV mAbs against chimeric VSV expressing MACV GPC (20). An additional limitation of this model is that both JUNV and MACV GPCs cannot use murine TfR1, which lacks a critical tyrosine 211 residue required for efficient binding (38). Instead, it is likely that a lower-affinity entry mechanism, such as binding to voltage-gated sodium channels (39, 40), or heparan sulfate proteoglycans (41), mediates virus attachment and entry. Cell-to-cell transmission, which has been documented for both arenaviruses and rhabdoviruses, may also play a role in viral dissemination (42, 43).

Several of the mAbs were produced as chimeras, combining the murine VH and VL regions with human IgG1 and Igκ constant regions to increase humanness. These modifications can reduce immunogenicity and antidrug antibody responses while improving the therapeutic potential in humans, especially for complement-mediated and ADCC responses. These humanized mAbs retained their binding and neutralizing capacities. The Hartley outbred guinea pig model of AHF with authentic JUNV challenge recapitulates many aspects of human disease and has been used to evaluate treatments with immune serum and mAbs (7, 15). Additionally, FcR-mediated ADCC function and complement fixation by humanized antibodies are maintained in this model (44). Here, we demonstrate that the newly discovered hu5B12 and hu1D5 antibodies are protective against lethal JUNV challenge in guinea pigs. In summary, we characterized a panel of mAbs raised against the JUNV glycoprotein that showed remarkable cross-reactivity, with some neutralizing JUNV potently and potentially having prophylactic and therapeutic value as humanized mAbs.

## MATERIALS AND METHODS

### Cells and viruses

VeroE6 cells (ATCC CRL-1586) were cultured in Dulbecco’s modified Eagle’s medium (Life Technologies), supplemented with 10% fetal bovine serum (FBS; HyClone), penicillin (100 units/mL), streptomycin (100 µg/mL) (P/S, Gibco), and buffer solution [1 M 4-(2-hydroxyethyl)-1-piperazineethanesulfonic acid (HEPES); Gibco] at 37°C and 5% CO_2_. Cultures were split every 2–3 days using trypsin (Thermo Fisher Scientific, Waltham, MA, USA). Sf9 cells were cultured in *Trichoplusia ni* medium formulation Hink (Gemini Bioproducts) containing 10% FBS and P/S, while BTI-TN-5B1-4 cells were grown in serum-free SFX insect cell medium (HyClone) supplemented with P/S.

Replication-competent recombinant rVSV-JUNV and rVSV-MACV, which express their respective arenavirus glycoproteins on the viral surface but lack the VSV G protein, were provided by Sean Whelan (Washington University, St. Louis). rVSV-JUNV and rVSV-MACV were expanded in VeroE6 cells for use in multiple experiments, including antibody characterization and efficacy testing in mice. VeroE6 cells (seeded the previous day) were infected with rVSV-JUNV or rVSV-MACV diluted in 1× minimal essential medium (MEM, Gibco) supplemented with P/S, HEPES, glutamine (200 mM L-glutamine, Gibco), and sodium bicarbonate (7.5% sodium bicarbonate solution, Gibco) and incubated at 37°C for 1 h. The virus was removed, and 1× MEM was added to the cells. After 24 h, the cytopathic effect was evaluated. The supernatant was collected after centrifugation at 4,000 × *g* and stored at −80°C for determination of infectious titer by classic plaque assay, as described below.

Viral stocks of Junín Candid#1 for *in vitro* neutralization assays were provided by Slobodan Paessler (University of Texas Medical Branch, Galveston) and expanded by infecting Vero cells at a multiplicity of infection (MOI) of 0.01. Virus-containing supernatants were collected 72 h post-infection, and cell debris was removed by centrifugation at 10,000 × *g* for 10 min at 4°C.

Codon-optimized, replication-competent, chimeric reporter viruses expressing the NW arenavirus GPC (VSVΔG-NWAV_GPC) were generated with the full GPC of JUNV/XJ13 (GenBank accession number AY358023.2), MACV/Carvallo (GenBank accession number NC005078.1), and CHAPV/810419 (GenBank accession number EU260463.1) replacing the VSV glycoprotein. These reporter viruses include enhanced green fluorescent protein (eGFP) under independent VSV promoter and terminator sequences upstream of VSV-N and were generated as previously described from plasmids provided by Lee et al. (45, 46). Reporter VSVΔG-NWAV_GPC viruses were rescued in HEK293T cells, expanded on VeroE6, and used for neutralization and antibody escape assays.

Junín strain Romero was expanded in VeroE6 cells at the Texas Biomedical Research Institute (Texas Biomed) at an MOI of 0.001 to generate challenge stocks for *in vivo* guinea pig experiments. Deep sequencing confirmed that the challenge stocks were wild-type Argentinian mammarenavirus, JUNV Romero strain (accession numbers JN801477.1 and JN801476).

### Plaque assay

Infectious titers of rVSV-JUNV and rVSV-MACV were determined in cell culture supernatant as PFU per milliliter. Confluent monolayers of VeroE6 cells were infected with 10-fold serially diluted cell culture supernatants, testing each dilution in duplicate at 37°C for 1 h. The virus was removed from cells, and the monolayers were overlaid with MEM containing 2% Oxoid agar (Oxoid purified agar, Thermo Scientific). Cells were then incubated for 24 h at 37°C, fixed with 3.7% paraformaldehyde for 1 h, and stained with crystal violet solution (Sigma-Aldrich). Finally, plaques were counted, and the viral titer was calculated. Infectious titers of VSVΔG-NWAV_GPC reporter viruses were determined by quantifying eGFP focus-forming units using a Tecan Spark Cyto cell imager 16 h after infection of Vero76 (ATCC CRL-1587) monolayers.

### Generation of mouse mAbs

Six- to eight-week-old female BALB/c mice (Jackson Laboratories) were immunized with 40 µg of DNA of pCAGGS plasmid encoding the complete open reading frame of the JUNV XJ13 strain glycoprotein (GenBank accession number ACO52428.1). The DNA plasmid was administered via an intramuscular electroporation method using the TriGrid delivery system from Ichor Medical Systems (47). After 3 weeks, a second immunization was performed, and after another 4 weeks, mice were boosted with 100 µg of recombinant JUNV GPC adjuvanted with 10 µg of poly(I:C). Three days post-boost, one mouse was euthanized, and the spleen was harvested for murine hybridoma fusion. The hybridoma fusion was performed as previously described (48). Briefly, splenocytes were washed and combined with SP2/0 myeloma cells at a 5:1 ratio; cells were then fused using polyethylene glycol (molecular weight, 4,000 g/mol) and grown on Clonacell-HY medium D (STEMCELL Technologies) for 10 days. Individual hybridoma colonies were picked and cultured in 96-well cell culture plates with medium E (STEMCELL Technologies) for 5 days. On the fifth day, clones were screened for reactivity against JUNV glycoprotein using an ELISA, as described below. Positive clones were isotyped using the Pierce Rapid Antibody Isotyping Kit (Life Technologies), and only IgG heavy-chain subclasses were selected for further expansion and purification with protein G sepharose 4 Fast Flow (GE Healthcare), as described previously in detail (48).

### Generation of humanized antibodies

For humanized antibody generation, sequencing was performed using SMARTer 5′ RACE technology (Takara Bio USA), adapted for antibodies to amplify variable genes from heavy and light chains for hybridomas expressing 1A2, 1D5, and 5B12. Briefly, RNA was extracted from hybridomas with the QIAGEN RNeasy Mini Kit (QIAGEN, Valencia, CA, USA), and first-strand cDNA was synthesized using isotype-specific constant gene 3′ primers, the SMARTer II A Oligonucleotide, and the SMARTscribe reverse transcriptase. cDNA amplification was achieved with SeqAmp DNA Polymerase (Takara), using a nested 3′ primer and a 5′ universal primer. The purified PCR products were subsequently Sanger sequenced with 3′ constant gene primers (Azenta/GeneWiz, South Plainfield, NJ, USA). Sequence data were blasted against the International ImMunoGeneTics Information System Human Germline Gene Database using V-Quest (http://imgt.org). DNA was synthesized by Twist Biosciences (South San Francisco, CA, USA) and cloned into the pTWIST-CMV-hIgG1 or pTWIST-CMV-hIgK expression plasmids. The heavy- and light-chain plasmids were transfected into Expi293F (Thermo Fisher Scientific) cells at a 1:1 plasmid ratio (wt/wt; a total of 0.8 mg/L of plasmids) using Polyethylenimine-MAX as the transfection reagent. Clarified, filtered supernatants (as above) were purified on Gore ProA membranes (PROA103; W. L. Gore & Associates, Newark, DE, USA) in Tris-buffered saline (pH 7.3) and eluted with 150 mM glycine and 40 mM NaCl (pH 3.0). The eluates were neutralized with 3 M Tris (pH 9.0) and assessed for monodispersity. Purified antibodies were dialyzed against phosphate-buffered saline (PBS) in a Slide-A-Lyzer (A52978, Thermo Fisher Scientific), filter-sterilized as above, and stored at −20°C.

### Recombinant arenavirus GPCs

The open reading frames encoding the ectodomain of the following arenavirus glycoproteins, JUNV (GenBank accession number ACO52428.1), MACV (GenBank accession number KM198592.1), SABV (GenBank accession number U41071.1), GTOV (GenBank accession number AY129247.1), and LASV (GenBank accession number GU481072.1), were expressed in the baculovirus expression system with a T4 trimerization domain, as previously described (19, 20). In brief, baculoviruses were generated in Sf9 cells, and the glycoproteins were expressed in BTI-TN-5B1-4 cells. Finally, the supernatant was purified via Ni^2+^-nitrilotriacetic acid as previously described (49).

NW arenavirus sGP1 (soluble glycoprotein 1) mammalian cell expression constructs (JUNV/Espindola, GenBank accession number OL774853.1; JUNV/Coronel, GenBank accession number OL774855.1; MACV/Chicava, GenBank accession number AY624355.1; and CHAPV/810,419, GenBank accession number EU260463.1) used for the Gyros immunoassay were synthesized by Twist Biosciences and cloned into a pTwist-CMV-BetaGlobin vector backbone using *Eco*RI and *Xba*I restriction sites. Constructs were transfected into Expi293F cells (A14527, Thermo Fisher Scientific), and cultures were clarified by centrifugation and filtration. Clarified supernatants were purified using the AKTA-PURE FPLC system (Cytiva Life Sciences, Marlborough, MA, USA) with the TALON Crude column (28953767, Cytiva Life Sciences). Elution fractions of NW arenavirus sGP1s were concentrated using a 10 kDa Amicon filter and further purified by size-exclusion chromatography (SEC) using the Superdex 10/300GL column (17517401, Cytiva Life Sciences). Resulting SEC elution fractions were re-concentrated with 10 kDa Amicon filters. JUNV Espindola-based GP1 mutants were generated with a single alanine substitution (or glycine if the natural sequence is alanine). Mutations were introduced into GP1 residues predicted to be in the receptor-binding domain.

### Enzyme-linked immunosorbent assay (ELISA)

For ELISA, 96-well plates (Immulon 4 HBX, Thermo Fisher Scientific) were coated with 2 µg/mL (50 µL/well) of the respective protein prepared in coating buffer (carbonate-bicarbonate buffer [KPL], pH 9.4) and incubated at 4°C. The next day, the plates were washed three times with PBS containing 0.1% Tween 20 (PBST) and blocked with 100 µL/well of 3% non-fat milk in PBST. After 1 h of incubation, 100 µL/well of various antibody dilutions, starting at 30 µg/mL and prepared in PBST with 1% non-fat milk, was added to the plates in duplicate. The plates were then incubated for 1 h at room temperature, followed by washing with 250 µL/well PBST three times. Antibody binding to the glycoprotein was detected by adding 100 µL/well of secondary antibody horseradish peroxidase-labeled anti-mouse IgG (GE Healthcare), diluted in PBST with 1% non-fat milk, and incubated for another hour at room temperature. The plates were washed with 250 µL/well PBST three times and developed by adding 100 µL/well of SigmaFast o-phenylenediamine dihydrochloride (Sigma-Aldrich) for 10 min. The reaction was stopped using 50 µL/well of 3 M hydrochloric acid (HCl), and the absorbance was determined at 490 nm via a Synergy 4 (BioTek) plate reader. A minimal binding concentration was defined as the final concentration at which the antibody signal remained greater than 3 standard deviations above the average of the blank wells. An irrelevant antibody, here referred to as negative, was used as a negative control, and an antihexahistidine antibody, here referred to as positive, was used as a positive control.

### Plaque reduction neutralization assay (PRNA)

Antibodies were fivefold serially diluted to a starting concentration of 100 µg/mL and mixed with 50 µL of virus at 1,500 PFU/mL; each dilution was tested in duplicate. The mixture was incubated at room temperature on a shaker for 1 h. Antibody-virus, virus-medium, or medium-only mixtures were then added to VeroE6 monolayers seeded the day before. After 1 h at 37°C, the inoculum was replaced with an agar overlay as described above. After 2 days of incubation at 37°C, the wells were fixed with 3.7% paraformaldehyde and stained with crystal violet. GraphPad Prism 11 was used to calculate IC_50_ values using logarithmic non-parametric regression, with a range from 0% to 100%. An irrelevant antibody, here referred to as negative, was used as a negative control.

### Antibody-dependent cellular cytotoxicity (ADCC) reporter assay

The ability of mAbs to trigger ADCC activity was tested with a commercial ADCC reporter assay kit (Promega) according to the manufacturer’s instructions. Briefly, 50,000 VeroE6 cells/well were seeded in white-bottom 96-well plates (Corning Costar) 1 day prior to the experiment. Cells were infected with rVSV-JUNV diluted in MEM at an MOI of 1 and incubated overnight. After 24 h, the medium was removed, and twofold serially diluted antibodies, starting at 30 µg/mL, were added, with each dilution tested in duplicate. Effector cells expressing murine FcγRIV receptor (for murine antibodies) or human FcγRIIIa receptor (for chimeric antibodies) were added. Plates were incubated for 6 h at 37°C. Bio-Glo luminescence reagent and substrate (Promega) were then added, and relative luminescence units were measured with the Synergy H1 Microplate Reader (BioTek). Finally, data were analyzed using GraphPad Prism 11.

### *In vivo* studies in Stat2^−/−^ mice

Six- to eight-week-old female and male Stat2^−/−^ C57BL/6 mice were mixed and randomized. Initially provided by Christian Schindler, the mice were bred at the Icahn School of Medicine at Mount Sinai. Groups of three mice were infected with 1,000 PFU of rVSV-JUNV via the intraperitoneal route, and organs were harvested on days 3 and 6 for viral titer determination. Each organ was homogenized using a BeadBlaster 24 (Benchmark) homogenizer, and the supernatant was used for plaque assays, as described above. Next, a prophylactic approach was used to assess whether the antibody confers protection against rVSV-JUNV infection. Each antibody group consisted of four mice and received 10 mg/kg of the antibody via the intraperitoneal route 2 h before infection with rVSV-JUNV. Mice were then infected with 1,000 PFU, and on day 3 post-infection, the spleen was harvested, and viral titer was determined by plaque assay, as described above.

### Gyros immunoassays

EZ-Link Sulfo-NHS-Biotin (Thermo Fisher Scientific) was used to biotinylate sGP1 proteins, which were then separated by size-exclusion chromatography to remove excess biotin using 7K MWCO Zeba Spin columns (Thermo Fisher Scientific). The Gyros technology platform (Gyros Protein Technologies, Uppsala, Sweden) was used to determine binding between sGP1 and mAbs on the Bioaffy 1000 HC Assay CD. Response units were detected using antihuman IgG F(ab′)2 (H + L) AlexaFluor 647 (Jackson ImmunoResearch Laboratories, West Grove, PA, USA).

### Biolayer interferometry

The mutational alanine scan was performed on an Octet RED384 (ForteBio; Sartorius, Göttingen, Germany). Samples were diluted and run in a buffer containing 10 mM HEPES, 150 mM NaCl, 3 mM ethylenediaminetetraacetic acid, 0.05% Tween 20, and 1 mg/mL bovine serum albumin (pH 7.4) and filter-sterilized with a 0.22 µm filter. mAb (1 µg/mL) was loaded onto antihuman IgG Fc capture biosensors (Sartorius). The regeneration buffer containing 10 mM glycine (pH 1.5) was used to regenerate biosensors as needed.

### VSV-NW arenavirus GPC neutralization

For the neutralization assays, Vero76 cells were plated in black, clear-bottomed 384-well plates treated with type 1 collagen (Corning, Corning, NY, USA), so that they were 75% confluent on the day of infection. mAbs were co-incubated with a target MOI of 0.2 focus-forming units (FFU) of the VSVΔG-NWAV_GPC chimeric virus for 1 h at 37°C, 5% CO_2_. The mAb-VSV cocktail was then added to the cells, which were incubated for 16 h at 37°C and 5% CO_2_. Supernatants were removed, and the cells were fixed in 4% paraformaldehyde containing Hoechst 33258 (Thermo Fisher Scientific) and imaged on a Tecan Spark Cyto (Tecan, Männedorf, Switzerland) to quantify FFU.

### *In vivo* studies in guinea pigs

To minimize pain and distress, euthanasia criteria were developed (labored/agonal breathing, being found prostrate/unresponsive, or exhibiting a total clinical score of 12 or greater, which results in immediate euthanasia). All euthanasia was approved by a veterinarian and performed using an overdose of sodium pentobarbital. Six-week-old female Hartley guinea pigs were purchased from Charles River Laboratories, implanted with radio-frequency identification and temperature transponders, and transferred to the animal biosafety level 4 laboratory following acclimatization. The day prior to challenge, subjects were treated by intraperitoneal injection of 10 mg of monoclonal antibody (or PBS for mock-treated subjects). The following day, subjects were anesthetized and inoculated intraperitoneally with 1,000 PFU of the Junín Romeo strain on day 0. For infections, 100 µL of virus stock diluted in sterile PBS was given to each animal. Temperature was monitored telemetrically throughout the study, and weight was recorded daily. Death and disease symptoms were recorded using the following definitions: discoordination, ataxia, transient seizures, or paralysis (hemiplegic or quadriplegic with the inability to reach the feeder or water bottle). Guinea pigs 3, 9, and 13 were removed from the study prior to infection (veterinarian decision based on findings during daily wellness checks).

### Escape experiment

A sigmoidal non-linear regression was performed in GraphPad Prism 11 to determine the 80% focus reduction neutralizing titer (FRNT80) of monoclonal antibodies against VSVΔG-JUNV/XJ13_GPC. The VSVΔG-JUNV/XJ13_GPC virus was incubated with 1× FRNT80 of each antibody for 1 h prior to addition to VeroE6 cells. Infected cells were monitored daily, and once significant eGFP expression and cytopathic effect were observed, the supernatants were clarified by centrifugation and used for the subsequent round of infection. The antibody concentration was doubled at each step over seven passages (up to 64× FRNT80). At 64× FRNT80 pressure, the virus was plaque-purified under an agarose overlay. Plaques were picked; RNA was extracted and used to synthesize cDNAs; the GPC region was amplified by PCR; and the amplicons were sequenced on an Illumina MiSeq. After quality control and adapter trimming and alignment to JUNV/XJ13_GPC, multiway pileups were used to determine single-nucleotide polymorphisms (SNPs) present in the viruses that escaped. No SNPs were observed during passage with a non-binding isotype antibody.

### Protein illustration with predicted binding sites

The glycoprotein sequence of JUNV GPC was obtained from GenBank accession number AAQ55251, and JUNV GP1 was sourced from the Protein Data Bank (PDB: 5W1K). Since there is no complete experimental structure of the full-length glycoprotein complex, the Chai-1 model (32, 33) was used to predict the structure of JUNV GPC in complex with the heavy and light chains of each antibody. The sequences of JUNV GPC and the antibody chains were used to generate their molecular structures, along with multiple sequence alignments from MMseqs2 and template data. A pocket restraint was incorporated to simulate amino acid substitutions in the protein, with chain A representing the protein and chain B representing the antibody. Residues in chain A within 5.0 Å of the antibody are predicted to be contact residues. Accuracy of the predictions was evaluated using pTM and ipTM scores. The structures were then aligned with JUNV GPC (PDB: 5W1K) using PyMOL (The PyMOL Molecular Graphics System, version 3.0; Schrödinger, LLC) or ChimeraX-1.6.1. The interactions between the antibody and JUNV GPC, along with contact mutations, were visualized using PyMOL.

### Statistical analysis

The IC_50_ value was determined by applying a non-linear regression to a two-parameter dose-response logistic curve, described by the equation *Y* = bottom + (top − bottom)/ (1 + (IC_50_/*X*)^Hill slope^). The fitted curve was overlaid on data points representing the mean of duplicate technical replicates, with error bars indicating the range. The area under the curve was calculated and reported along with the 95% CI. Survival curves, as shown in Fig. 4, were analyzed using the Mantel-Cox log-rank test. Changes in body weight and body temperature are expressed as percentages, with mean values and standard deviation error bars derived from six subjects, except in cases where survival decreased the sample size.

## Data Availability

Data are available per reasonable request from the corresponding author.

## SUPPLEMENTAL MATERIAL

Supplemental Material mbio.00214-26-s0001.pdf Supplemental figure and tables.

ASM does not own the copyrights to Supplemental Material that may be linked to, or accessed through, an article. The authors have granted ASM a non-exclusive, world-wide license to publish the Supplemental Material files. Please contact the corresponding author directly for reuse.
