# Supplementary material for: Monoclonal antibodies raised against the Junín virus glycoprotein show broad reactivity and provide protection in vivo
Source: mBio. 2026 Sep 25;17(10):e00214-26. doi: 10.1128/mbio.00214-26 (PMC13712815; doi:10.1128/mbio.00214-26)
Supplement: Supplementary file 1 [file mbio.00214-26-s0001.pdf]

Supplemental material for:

Monoclonal antibodies raised against the Junín virus glycoprotein show broad reactivity and provide protection *in vivo*

*Garazi Pena Alzua[1,2], Sharon Jan[3], Fatima Amanat[1], Grecia A. Sandoval[3], Christopher A Sumner[3], Stephen Won[3], Anna Fisher[3], Anupama Sinha[3], Brooke N. Harmon[3], Elizabeth B Allmon[4], Kendra J Alfson[4], Yenny Goez-Gazi[4], John W Dutton III[5], Ricardo Carrion Jr[4], Sneha Rangarajan[6], Melissa C Kerzic[6], Carter A Mitchell[6], Chris M Cirimotich[7], Dylan M Johnson[4,8], Florian Krammer[1,2,9,10,11]**

[1]Department of Microbiology, Icahn School of Medicine at Mount Sinai, New York, New York, USA
[2]Center for Vaccine Research and Pandemic Preparedness (C-VaRPP), Icahn School of Medicine at Mount Sinai, New York, NY, USA
[3]Department of Biotechnology & Bioengineering, Sandia National Laboratory, Livermore, California, USA
[4]Texas Biomedical Research Institute, 8715 W. Military Dr., San Antonio, Texas 78227, USA
[5]Southwest National Primate Research Center, Texas Biomedical Research Institute, 8715 W. Military Drive, San Antonio, Texas 78227, USA
[6]Kemp Proteins, LLC, 5119 Pegasus Ct. St N, Frederick, Maryland 21704 USA
[7]Battelle Biomedical Research Center, 1425 State Route 142, West Jefferson, Ohio 43162 USA
[8]Department of Biosecurity & Bioassurance, Sandia National Laboratory, Livermore, California, USA
[9]Department of Pathology, Molecular and Cell Based Medicine, Icahn School of Medicine at Mount Sinai, New York, NY, USA
[10]Ignaz Semmelweis Institute, Interuniversity Institute for Infection Research, Medical University of Vienna, Vienna, Austria
[11]Ludwig Boltzmann Institute for Science Outreach and Pandemic Preparedness at the Medical University of Vienna, Medical University of Vienna, Vienna, Austria

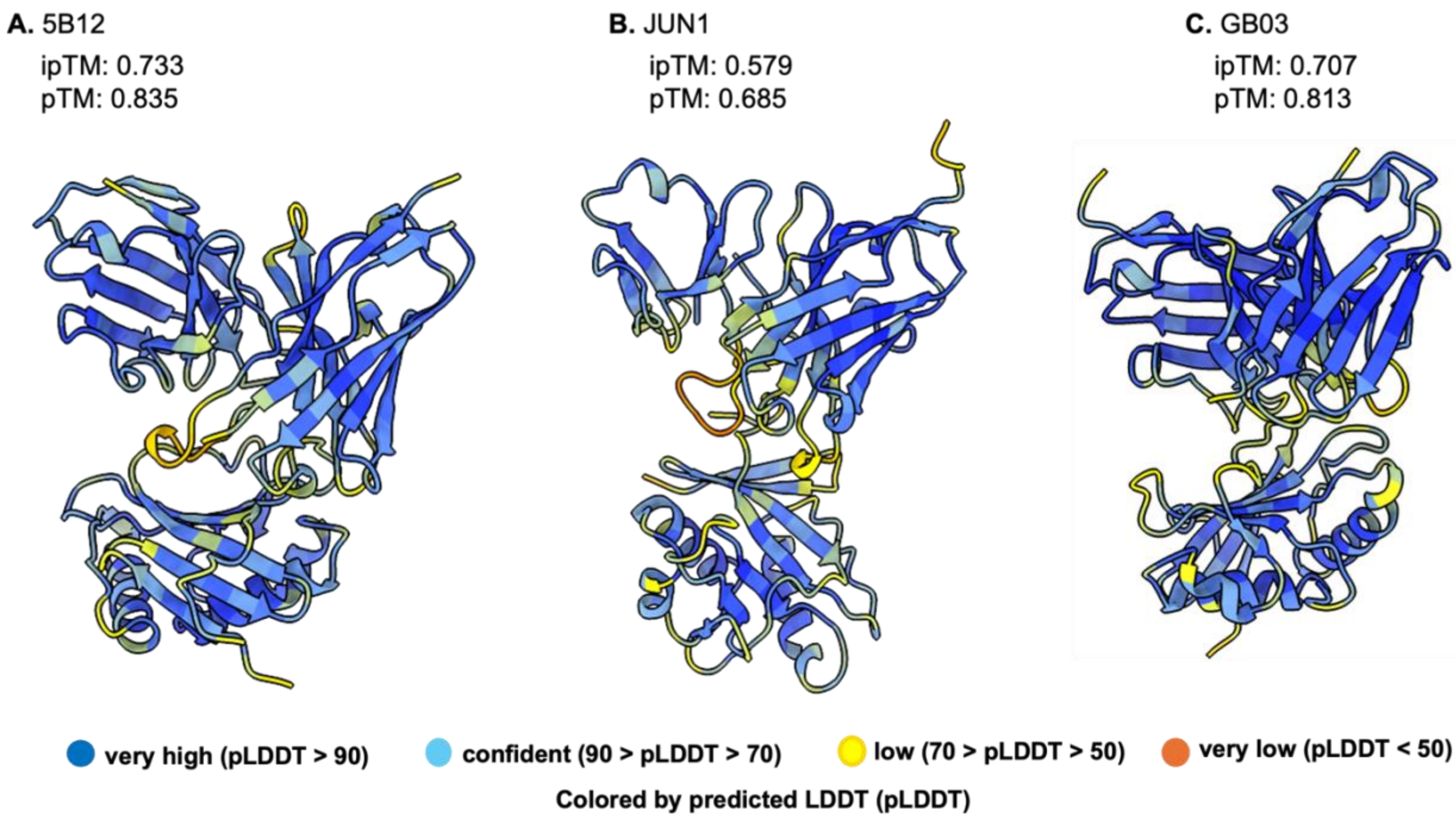

Supplementary Fig. 1. Prediction structures and scores. Chai Discovery was used to predict the structure of the JUNV GP1 (PDB:5W1K) in complex with the heavy and light chain sequences of (A) 5B12, (B) JUN1, and (C) GB03 antibodies. Chai Discovery-generated files were used in ChimeraX to illustrate the models. Color is based on the AlphaFold-predicted local distance difference test (pLDDT) to provide an estimate of the accuracy of the protein prediction. Blue indicates a good prediction, and orange indicates a less confident prediction. On top of each model, the interface predicted template modeling (ipTM) and template modeling (pTM) scores are shown.

Table S1. Antibody variable region sequences.

Name	Backbone	VH or VL amino acid sequence
1A2	HuIgG1	QVQLQQSGAELVRPGVSVKISCKGSGYTFTDYPMHWVKQSHAKSLEWIGIINTYYGDTTYNQKFKGKATMTVDKSSSTAYMELARLTSEDSAIYYCVRWDYYGGTYEDYWGQGTTLTVSS
1A2	HuIgκ	GQIVLTQSPAIMSASPGERVTMTCSATSSIGYMHWYQQTPGTSPKRWIYDTSKLASGVPARFSGSGSGTYYSLTISSMEAEDGATYYCHQRTSYPYTFGGGTKLEI
1D5	HuIgG1	QVQLQQSGAELVRPGVSVKISCKGSGYTFTDYPMHWVKQSHAKSLEWIGIINTYYGDTTYNQKFKGKATMTVDKSSSTAYMELARLTSEDSAIYYCVRWDYYGGTYEDYWGQGTTLTVSS
1D5	HuIgκ	GQIVLTQSPAIMSASPGERVTMTCSATSSIGYMHWYQQTPGTSPKRWIYDTSKLASGVPARFSGSGSGTSYSLTISSMEAEDAATYYCHQRTSYPYTFGGGTKLEI
5B12	HuIgG1	QVQLQQSGAELVRPGVSVKISCKGSGYTFTDYAMHWVKQSHAKSLEWIGIINTYYGDTTYNQKFKGKATMTVDKSSSTAYMELARLTSEDSAIYYCARWDYFGGNYEDYWGQGTTLTVSS
5B12	HuIgκ	GQIVLTQSPAIMSASPGEKVTMTCSASSSISYMHWYQQKPGTSPRRWIYDTFRLSSGVPARFTGSGSGTSYSLTISSMEAEDAATYYCHQRTSYPYTFGGGTKLEIK
GB03	HuIgG1	EVQLVESGGGLVQPGGSLKLSCAASGFSFSNCGMSWVRQTPDRRLELVAAINRDGDITYHPDSVKGRFTISRDNAKNTLYLQMSSLKSEDTAMYYCAREDYYGLYFYVMDYWGQGTSVTVSS

GB03	HuIgκ	QIVLTQSPAIMSASPGEKVTITCSASSIVSYMHWFQQKPGTSPKLWIYGTSNLASGVPARFSGSGSGTSYSLTISRMEAEDAATYYCQQRNSYPPTFGGGTKLEIK
GD01	HuIgG1	EVKLQQSGAELARPGTSVKLSCKASGYTFTNYWMQWIKQRPGQGLEWIGAVYPGDGDTRFSQKFKGKATLTADKSSSTAYMQLSSLSSEDSAVYFCARRRVYYGSNYIYALDYWGQGTSVTVSA
GD01	HuIgκ	GDIVMTQSQKFMSTSIGDRVSITCKASQNVGSAVAWYQQKPGQSPKLLIYSASNRYTGVPDRFIGSESGTDFTLTISNMQSEDLADYFCQQYSSYPLAFGAGTKLELK
JUN1	HuIgG1	QIQLVQSVETGGGLVRPGNSLKLSCVTSGFTFSNYQMHWLRQPPGKRLEWIAVITVKSDNYGANYVESVKGRFAISRDDSKSSVYLEMNRLREEDTATYFCSRSGIYDGYYAYAMDYWGQGTSVTVSS
JUN1	HuIgκ	GSVVMTQSQKFMSTSVGDRVSITCKASQIVGTSVAWYQQKAGQSPKLLIYWASTRHTGVPDRFTAGGSGTDFTLTITNVQSEDLADYFCQQYATYPLTFGSGTKLELK
OD01	HuIgG1	EVQLQQSGTVLARPGASVKMSCKASGYTFTSYWMHWIKQRPGQGLEWIGAIYPGDSDTKYNQKFKGKAKLTAVTSTSTAYMELSSLTNEDSAVYYCTRRNTLTGDYFDYWGQGTTLTVSS
OD01	HuIgκ	GDIVLTQSPASLAVSLGQRATISCRASESVDDYGISFMNWFQQKPGQPPKLLIYTASSQGSGVPARFSGSGSGTDFSLNIHPMEEDDTAMYFCQQSKEVPYTFGGGTKLEIK

Table S2. Amino acid changes in JUNV GP1

	Amino acid sites that reduce binding to JUNV GP when replaced with alanine		Amino acid sites evolved under selective pressure	
mAbs name	position	change	position	change
hu5B12	165	R-A	172	G-D
	169	K-A	218	Q-K
	223	P-A		
huGB03	173	F-A	125	I-T
	214	L-A	126	I-V
	223	P-A		
huJUN1	121	Q-A	169	K-T/M
	122	Y-A	170	T-I
	170	T-A		
	173	F-A		
	223	P-A		
huOD01	111	S-A		
	112	F-A		
	114	D-A		
	115	I-A		
	124	V-A		
	135	I-A		
	167	R-A		
	170	T-A		
	212	L-A		

Table S3. Structure prediction results

		Accuracy score with full-length glycoprotein		Accuracy score with GP1 structure	
mAbs	antibody chain	pTM	ipTM	pTM	ipTM
5B12	heavy	0.72	0.58	0.81	0.54
	light	0.65	0.27	0.72	0.28
JUN1	heavy	0.64	0.27	0.67	0.31
	light	0.66	0.23	0.78	0.29
GB03	heavy	0.64	0.28	0.66	0.25
	light	0.66	0.42	0.72	0.33
